# New Insights in the Genetics and Genomics of Adrenocortical Tumors and Pheochromocytomas

**DOI:** 10.3390/cancers14041094

**Published:** 2022-02-21

**Authors:** Peter Igaz

**Affiliations:** 1 Department of Endocrinology, ENS@T Research Center of Excellence, Faculty of Medicine, Semmelweis University, H-1083 Budapest, Hungary; igaz.peter@med.semmelweis-univ.hu; Tel.: +36-1-266-0816; 2 Department of Internal Medicine and Oncology, Faculty of Medicine, Semmelweis University, H-1083 Budapest, Hungary; 3 MTA-SE Molecular Medicine Research Group, Eötvös Loránd Research Network, H-1083 Budapest, Hungary

This article collection includes 16 scientific papers that present the current state of the art of genetics and genomics research in the fascinating field of adrenal tumors. In recent years, significant advancements in genetics, epigenetics and genomics have been made, and in our Special Issue, several of these issues are presented by international leaders of the field.

Adrenal tumors include adrenocortical and adrenomedullary tumors. Among adrenocortical tumors, hormonally inactive, benign adenomas are the most common, but hormone secretion is associated with significant morbidity and mortality. Primary aldosteronism represents the most frequent hormonally active adrenocortical tumor syndrome that is a common cause of secondary hypertension, being responsible for 5–10% of all cases. Primary aldosteronism is almost invariably caused by a benign, unilateral adenoma or bilateral hyperplasia. Malignant adrenocortical cancer (ACC) is rare and has a poor prognosis; moreover, its management is difficult due to difficulties in diagnosis and treatment. Adrenomedullary tumors, pheochromocytomas (and their extra-adrenal counterparts, i.e., paragangliomas) are also associated with significant morbidity and mortality due to severe hypertension, cardiovascular complications and, in some cases, metastatic disease.

The manuscripts in this Special Issue discuss the genetic–epigenetic–genomic issues of three major adrenal-tumor-related diseases: primary aldosteronism, adrenocortical cancer and pheochromocytoma/paraganglioma.

Recent studies have shown that a significant proportion of aldosterone-producing adrenocortical adenomas harbor somatic mutations in a subset of genes mostly coding ion channels. The disease phenotype might be associated with some genetic variants, as the article by Chang et al. shows greater recovery from arterial stiffness in adenomas harboring *KCNJ5* somatic mutations [1]. The molecular features of a novel mutation in the *ATP2B3* gene are presented in detail in the article by Liao et al. [2]. By using advanced bioinformatics approaches, Gong et al. characterized the metabolome and tissue microenvironment in aldosterone-producing adenomas and showed metabolic reprogramming toward fatty acid β-oxidation and glycolysis; moreover, an immunosuppressive tissue microenvironment was also found [3]. In a systematic review, Spyroglou et al. present recent developments in the transcriptomics, epigenetics and metabolomics of primary aldosteronism [4]. MicroRNAs belonging to the group of non-coding RNA molecules are also investigated as circulating markers in primary aldosteronism [5].

The diagnosis of adrenocortical cancer is challenging. There is no available preoperative, bloodborne marker of malignancy, but even the histological diagnosis of malignancy is often difficult. Moreover, prognostic markers are warranted to aid in the clinical management of patients. Artificial-intelligence-based approaches can be used to uncover novel markers, as presented in two studies of this Special Issue. Marquardt et al. revealed novel transcriptomic markers with prognostic relevance [6], whereas in the study by our research group (Turai et al.) microRNA combination markers for adrenocortical malignancy with high diagnostic performance are presented [7]. A peculiar feature of adrenocortical cancer is represented by the high incidence of pediatric ACC in southern Brazil due to a founder mutation in the *TP53* gene. Prognostic factors, newborn screening, surveillance and treatment costs are discussed in the article by Tosin et al. [8]. The treatment of adrenocortical cancer is also problematic, as mitotane is the only available adrenal-specific drug with several side effects and a narrow therapeutic range. Moreover, its mechanism of action is only partially elucidated. In their review article, Lo Iacono present in vitro data of mitotane action highlighting controversial issues [9].

Pheochromocytoma/paraganglioma (PPGL) is associated with the highest heritability among human tumors, as about 40% of tumors are associated with germ-line mutations of susceptibility genes. Recent advancements in the genetics of PPGL are presented by Jhawar et al. [10], and the relevance of genetic findings in the clinical management of PPGL patients is detailed in the review by Flores et al. [11]. The molecular methods and their analytical performance for genetic diagnosis are crucial, as presented by Sarkadi et al. [12]. 

The diagnosis of PPGL malignancy is challenging, as there is no reliable histological or blood-borne marker for metastatic disease. A new metastasis risk gene, NOP10, which is related to telomere maintenance, has been reported by Monteagudo et al. [13], and the potential for non-coding RNA markers is presented in our review [14].

In an interesting original study by Canu et al. on a large Italian cohort, the increased incidence of various secondary malignancies in non-syndromic PPGL patients is documented [15]. This observation is relevant regarding the surveillance and follow-up strategy for PPGL patients. 

Alterations in the mitochondrial respiratory chain and redox balance are major pathogenic factors in a subgroup of PPGL. The article by Dona et al. presents a novel zebrafish model that could be efficiently used for the study of PPGL [16].

I do hope that the articles included in this Special Issue will be helpful for the readers to gain an insight into this rapidly involving and dynamic field of endocrine oncology.
